# Synthetic RNA Silencing of Actinorhodin Biosynthesis in *Streptomyces coelicolor* A3(2)

**DOI:** 10.1371/journal.pone.0067509

**Published:** 2013-06-27

**Authors:** Gabriel C. Uguru, Madhav Mondhe, Shan Goh, Andrew Hesketh, Mervyn J. Bibb, Liam Good, James E. M. Stach

**Affiliations:** 1 School of Biology, Newcastle University, Newcastle upon Tyne, United Kingdom; 2 Department of Pathology and Infectious Diseases, Royal Veterinary College, University of London, London, United Kingdom; 3 John Innes Centre, Norwich, United Kingdom; Center for Genomic Regulation, Spain

## Abstract

We demonstrate the first application of synthetic RNA gene silencers in *Streptomyces coelicolor* A3(2). Peptide nucleic acid and expressed antisense RNA silencers successfully inhibited actinorhodin production. Synthetic RNA silencing was target-specific and is a new tool for gene regulation and metabolic engineering studies in *Streptomyces*.

## Introduction

Bacteria use endogenous transcripts to help regulate a diverse range of cellular processes. These RNAs include riboswitches (mRNA leaders that affect transcription in *cis*), protein-binding small RNAs, CRISPR RNAs (involved in the targeting and degradation of foreign DNA) and both *cis*- and *trans*-encoded mRNA-binding sRNAs [1]. The application of computational searches in combination with RNA sequencing has enabled prediction of hundreds of putative regulatory RNAs in multiple species; the verified sRNAs of *Escherichia coli* account for ≈ 2% of its identified genes [2]. Regulatory RNAs can act as repressors or activators of transcription, or as stabilizers of target transcripts, and are involved in a wide variety of cellular processes including virulence regulation, quorum sensing, stress responses and secretion [3].

The genus *Streptomyces* includes species that have complex genetic regulatory pathways, in part due to a need for morphological differentiation and the production of diverse bioactive secondary metabolites, including the majority of known antibiotics [4]. The biotechnological importance of this genus makes advances in the understanding and manipulation of regulatory RNAs of interest. Accordingly, bioinformatics [5], [6], [7] and deep-sequencing [8] have been applied to identify putative small RNAs in the genomes of *Streptomyces* species. In a recent study, D’Alia *et al*. [9] were the first to elucidate the regulatory effect of a *cis*-encoded antisense sRNA, cnc2198.1 in glutamine synthetase I. Expression of cnc2198.1 in *Streptomyces coelicolor* A3(2) resulted in decreased growth, reduced protein production and synthesis of the red-pigmented antibiotic, undecylprodigiosin. A second example of a *cis*-encoded sRNA *α-abe*A was identified in *S. coelicolor* as part of a four-gene cluster involved in the enhanced production of the blue-pigmented antibiotic, actinorhodin, although the exact role of *α-abe*A has yet to be elucidated [4], [6]. *Trans*-encoded sRNAs are analogous to eukaryotic miRNAs, and, in many cases, require an RNA chaperone to mediate regulation [1]. *In vitro* and *in silico* evidence of a *trans*-encoded antisense sRNA *mic*X, a putative activator of actinorhodin biosynthesis, has been reported [10], [11] with a recent study providing *in vivo* evidence for a *S. coelicolor trans*-encoded sRNA (scr5239) acting as a repressor of extracellular agarase expression [12]. Both *cis*- and *trans*-encoded sRNAs are thought to regulate gene expression as antisense RNA silencing molecules (asRNAs) that bind to their mRNA target, inhibit ribosome access and/or trigger mRNA degradation [13].

The studies cited above demonstrate that *Streptomyces* species use sRNAs to regulate gene expression; as such it is attractive to consider ways to exploit these molecules in practical applications. The use of conditional antisense RNA silencing may be of use not only in the elucidation of secondary metabolite regulation, but also in studies where gene knockouts are unsuitable e.g. when monitoring the affect of transcript abundance on gene expression; determining the minimally required levels of expression of essential genes [14]; and where the physical structure of the chromosome is related to transcriptional activity [15]. Synthetic RNA silencing, here defined as the use of antisense sequences that are either non-biological in origin or *trans*-acting RNAs that are not naturally expressed by the host, has yet to be demonstrated in the genus *Streptomyces*. Oligo-nucleobase antisense RNA silencers, such as peptide nucleic acid (PNA) and phosphorodiamidate morpholino oligomers (PMO), are DNA mimics that maintain naturally occurring DNA bases but have non-natural linkages between bases. PNAs and PMOs, conjugated to peptide carrier molecules, have been used to silence gene expression in a number of Gram-positive and Gram-negative species [16], [17], [18], and have the advantage of being resistant to biological degradation and do not require genetic transformation for delivery; carrier peptides attached to the PNA mediate delivery across the cell wall/membrane. Expressed RNAs designed to hybridize to target mRNA have been applied successfully in both Gram-positive and Gram-negative species, with Gram-positive species initially yielding more consistent gene silencing [19]. Recently, the addition of paired-termini (antisense RNA flanked by inverted repeats) proved successful in improving the efficacy of antisense RNA gene silencing in *E. coli*, most likely through the stabilization, and thereby increased abundance, of the asRNA [20].

In this study, we demonstrate, for the first time, the successful application of synthetic RNA silencing in *Streptomyces*. Both peptide-PNA conjugants and paired-termini antisense RNA were used to silence the expression of a gene required for actinorhodin biosynthesis. The development of antisense RNA gene silencers for *Streptomyces* species provides complementary tools to conventional genetics for the elucidation of regulatory pathways and gene function and will be a valuable tool in metabolic engineering.

## Results and Discussion

### Peptide-PNA Gene Silencing of actI-ORF1

**Figure 1:**
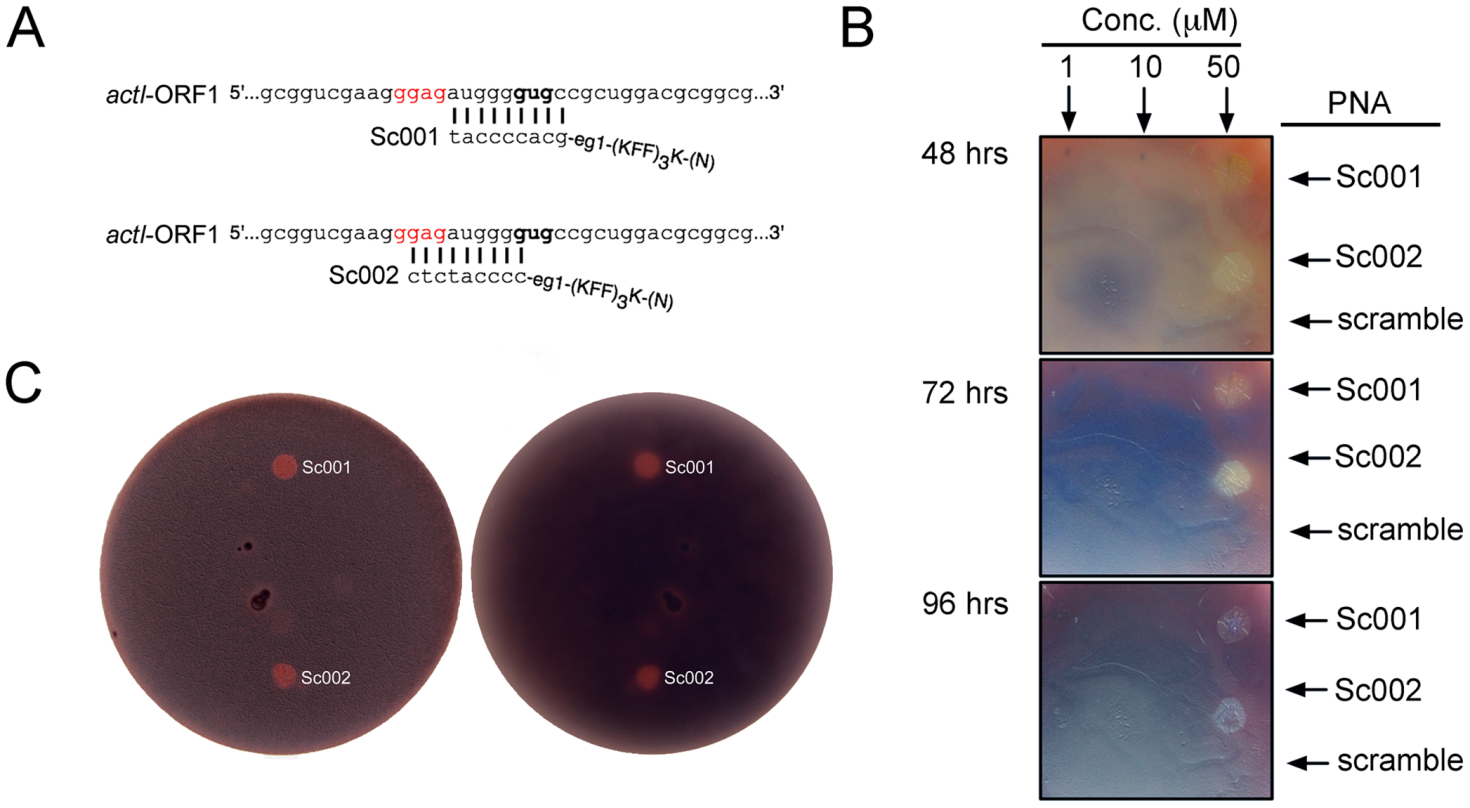
RNA silencing using peptide-PNA. (A) Schematic diagram representing the binding site of antisense peptide-PNAs designed to prevent transcription of *act*I-ORF1. The start codon of *act*I-ORF1 is shown in bold, the putative Shine-Dalgarno site in red. Sc001 and Sc002 differ in the degree of coverage of the start codon. (B) Demonstration of the ability of peptide-PNAs to silence production of actinorhodin in *S. coelicolor*. Peptide-PNA solutions were applied to a lawn of *S. coelicolor* (after 24 hrs) on MPCA agar and incubation continued for a further 72 hrs. Repression of actinorhodin production is clearly visible with 50 µM treatment using either Sc001 or Sc002; no reduction in actinorhodin production was evident when a scramble-PNA with limited complementarity to the *S. coelicolor* genome was used at the same concentration. (C) Peptide-PNA solutions were applied directly to a lawn of *S. coelicolor* MT1110 on ISP-4 agar and were photographed from on top (left) and from bottom (right) after 96 hrs incubation at 28°C.

To investigate the use of synthetic RNA gene silencers, we targeted the actinorhodin polyketide beta-ketoacyl synthase subunit gene (*actI*-ORF1). Two peptide-PNAs (Sc001 and Sc002) were designed and applied as antisense agents targeting the translation initiation region of *act*I-ORF1 (Fig. 1A). Guidelines for the design of peptide-PNA antisense gene silencers have been reported elsewhere [21], [22]. In this study we used 9 bp PNAs as they are reported to have improved uptake properties; longer PNAs also have an increased risk for non-target binding and self-complementarity [35]. In PNAs of 10–15 bp, mismatches with the target of >2 bp abolish activity, while mismatches of 1 bp abolish or significantly reduce activity. Thus, for the PNAs used in this study, it is likely that only genes with perfect matches in the target region will be significantly repressed. We identified all potential off-target genes for Sc001 and Sc002 (19 and 33 targets, respectively [Table S1 information]), none of which are predicted to have a role in antibiotic synthesis (many are annotated as possible or hypothetical proteins). For Sc001 only the intended target has a perfect match with the PNA, while Sc002 matches the target and SCO6703, a gene predicted to encode a putative 3-oxoacid CoA-transferase that is possibly involved in aromatic acid degradation. While a role for SCO6703 in actinorhodin production cannot be ruled out, it is unlikely that repression of this gene would play a significant role in the peptide-PNA mediated silencing of actinorhodin biosynthesis (Sc001 has 4 bp mismatches with the TIR of SCO6703 and results in a similar phenotype to Sc002). Actinorhodin production was clearly inhibited at 50 µM for both Sc001 and Sc002, but not by a scrambled peptide-PNA (Fig. 1B). The viability of subcultures taken from the area of peptide-PNA application and the lack of inhibition by the scrambled peptide-PNA indicates that the silencing of actinorhodin production was due to gene silencing and not growth inhibition. Experiments using ISP-4 agar medium indicated that neither PNA affected production of undecylprodigiosin (Fig. 1C). These results show that *S. coelicolor* is accessible to synthetic RNA silencing. Furthermore, susceptibility to PNA mediated gene silencing suggests that other RNA silencing strategies may also be effective in this genus. As PNA synthesis is relatively expensive, we also evaluated synthetic RNA silencing in *S. coelicolor* using expressed antisense RNA.

### Expressed Antisense RNA Silencing of actI-ORF1

**Figure 2:**
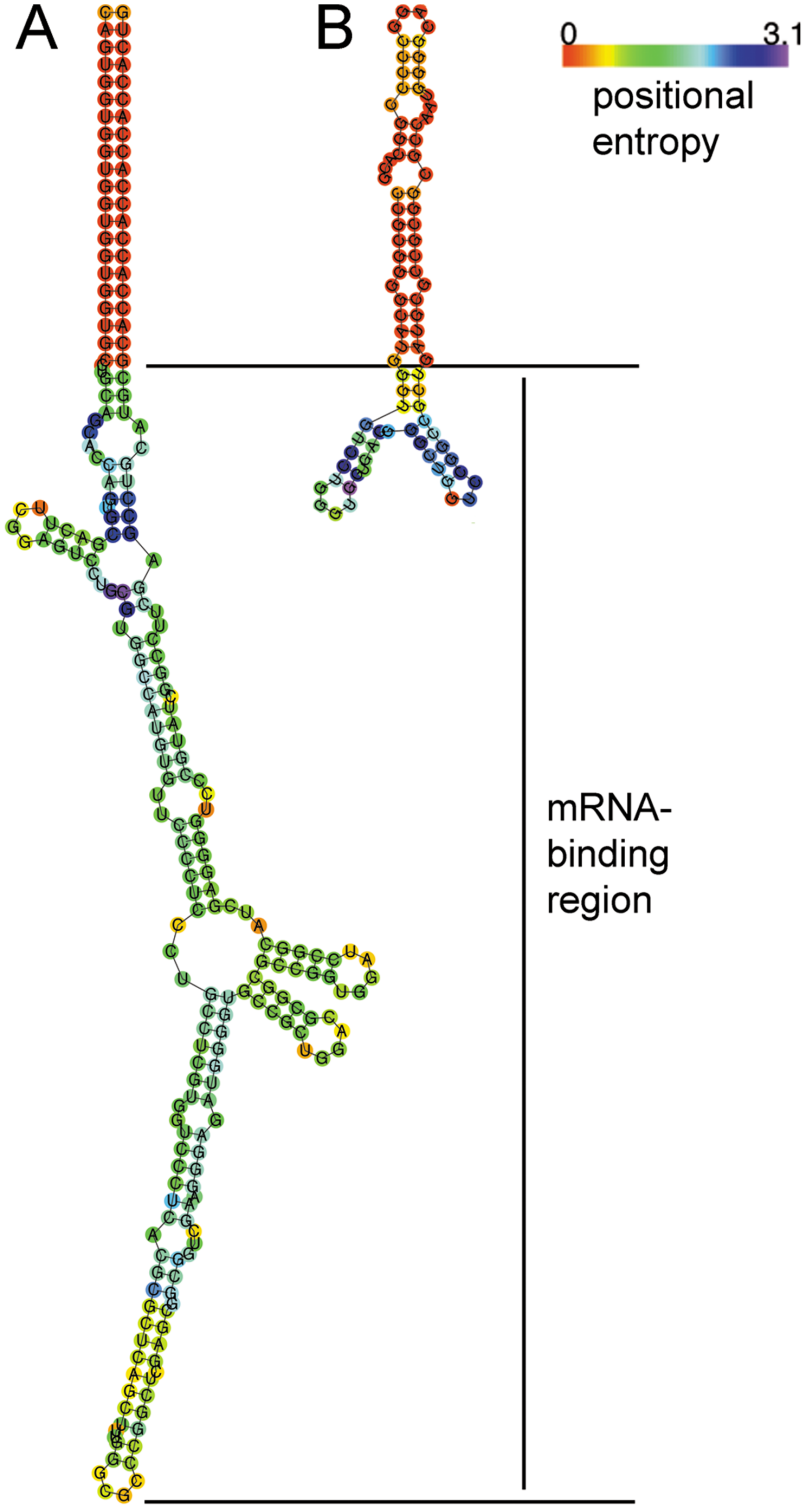
Predicted secondary structures of antisense RNAs. Secondary structure of (A) the paired-termini antisense *act*I-ORF1 asRNA (−92 to +63 bp) designed in this study for silencing of actinorhodin production, and (B) *mic*X, a naturally occurring *trans*-encoded antisense RNA from *Streptomyces lividans* involved in the activation of actinorhodin biosynthesis. The *mic*X asRNA has a predicted paired-end structure which suggests that the synthetic strategy followed in this study (addition of paired termini to antisense RNA) may be analogous to natural antisense mechanisms in *Streptomyces*. *mic*X also contains non-complementary nucleotide sequences in the paired region which may serve to protect antisense RNAs from RNase III cleavage.

**Figure 3:**
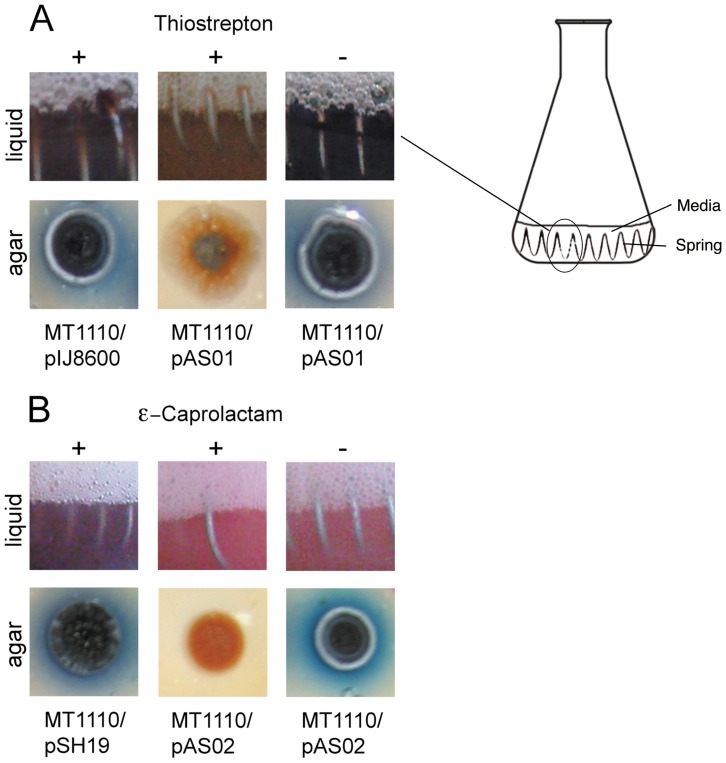
Actinorhodin production in *S. coelicolor* MT1110 and effects of RNA silencing. (A) MT1110/pAS01 after 6 days of growth in R5 liquid medium (top panels, the region shown is represented by the flask on the right) and R5 agar (bottom panels) containing thiostrepton (+ induction) or DMSO (- induction). MT1110/pIJ8600 served as a control. (B) As above with MT1110/pAS02, ε-caprolactam as the inducer of expression and MT1110/pSH19 as the control.

DNA sequences (120–160 bp) covering the 5′UTR, RBS and >50 bp of the coding region of *act*I-ORF1 were examined as potential antisense RNAs. Sequences with low secondary structure, and high positional entropy in the mRNA binding portion of the asRNA, were identified using RNAfold [23] (Fig. 2). A 155 bp region (-92 to +63) with the desired features was PCR-amplified from *S. coelicolor* MT1110 gDNA using primers with 21 bp inverted repeat overhangs, designed to generate antisense RNA transcripts with paired termini (PTasRNA) [20]. This amplicon was cloned into integrative pIJ8600 [24] and replicative pSH19 [25] vectors to form pAS01 and pAS02, respectively. Both pAS01 and pAS02 were used to transform *S. coelicolor* MT1110 and actinorhodin production was monitored in a number of media. For liquid and agar R5 media, both *S. coelicolor* MT1110/pAS01 and pAS02 showed visible reduction of actinorhodin production when induced with thiostrepton and ε–caprolactam, respectively (Fig. 3). In liquid culture, actinorhodin production was clearly reduced in non-induced MT1110/pAS02 indicating that, in our hands, expression of antisense RNA from vector pSH19 was likely to occur in the absence of induction. For this reason, pAS02 was excluded from further analysis.

### Quantification of the Effects on Antibiotic Production

**Figure 4:**
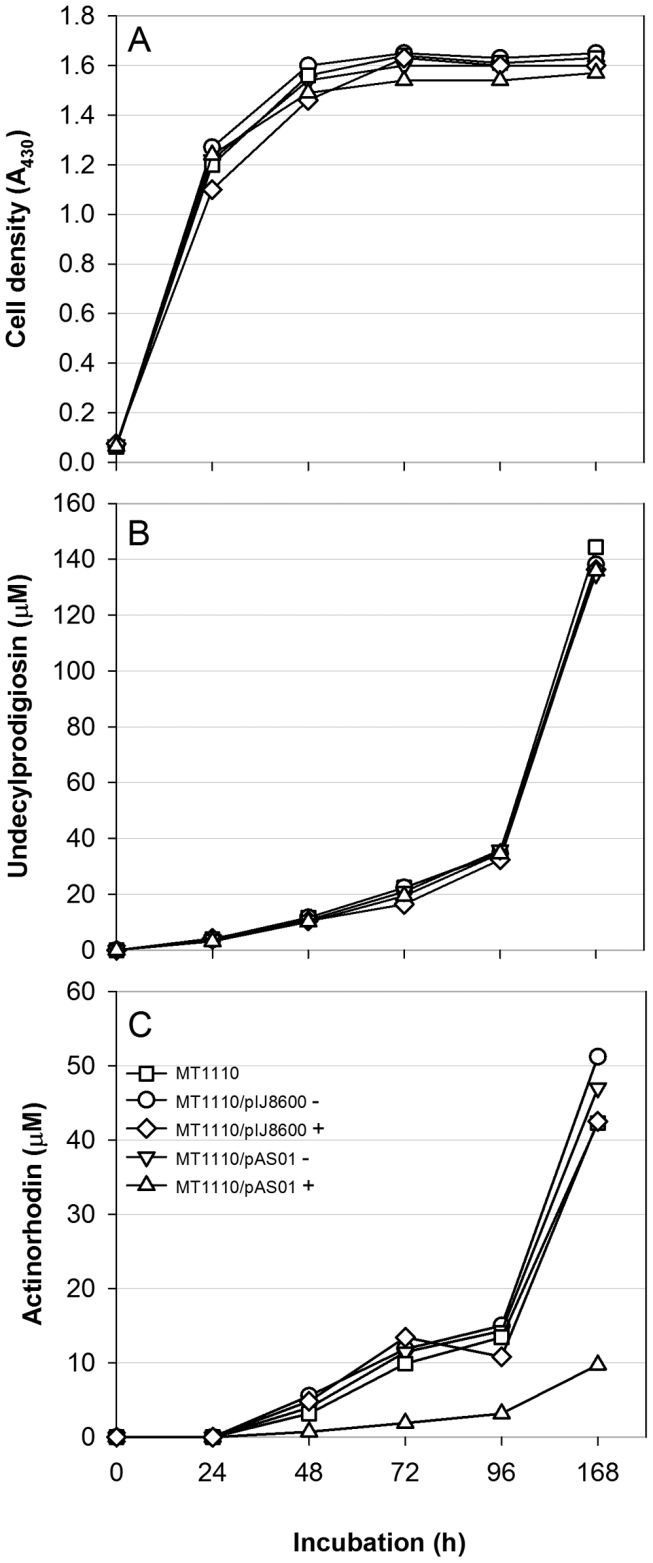
Antisense mediated decrease in actinorhodin production. *S. coelicolor* MT1110 strains were grown in R5 liquid medium containing thiostrepton (+ induction) or DMSO (- induction) for 5 days. (A) Cell growth, and (B) undecylprodigiosin production were similar in all cases. (C) Actinorhodin production was reduced when antisense RNA was induced (MT1110/pAS01+). The data represent the average of three independent determinations.

To determine whether RNA silencing using PTasRNA was specific to *actI*-ORF1, growth and antibiotic production were monitored for MT1110/pAS01 and control cultures in R5 liquid medium (Fig. 4). In all cases, growth and the production of undecylprodigiosin was equivalent to that of uninduced *S. coelicolor* MT1110, indicating that expression of the antisense RNA was not growth inhibitory, and that reduced antibiotic production was restricted to actinorhodin biosynthesis (production of the calcium-dependent antibiotic was also unaffected by antisense RNA production, Figure S2). In contrast, induced MT1110/pAS01 displayed unaltered growth but ≈ 4-fold reduced actinorhodin production; 10 µM for induced cultures, compared to >40 µM for uninduced MT1110/pAS01 and other controls after 7 days incubation (Fig. 4).

### Quantification of the Effect on actI-ORF1 Transcription

**Figure 5:**
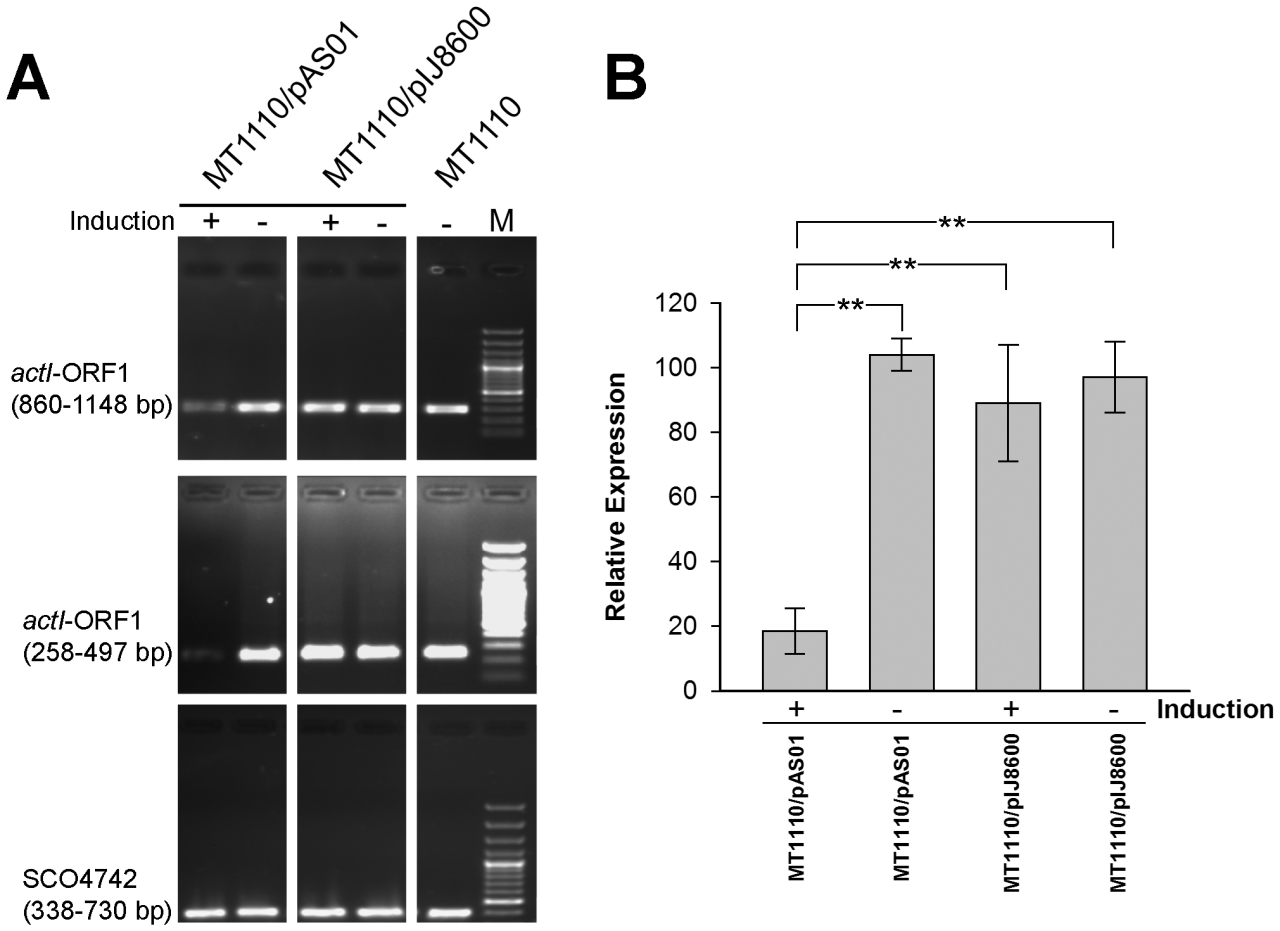
Effect of expressed antisense RNA on *act*I-ORF1 mRNA levels. RT-PCR of *act*I-ORF1 from RNA extracted from MT1110 strains grown for 3 days in R5 liquid medium that contained thiostrepton (+) or DMSO (-) as a control. Two primers sets (top and middle panel) designed to amplify different regions of the *actI*-ORF1 mRNA were used, with SCO4742 (bottom panel) serving as an internal control. M = 100 bp DNA ladder. (B) qRT-PCR of *act*I-ORF1; SCO4742 served as the internal reference and expression was calculated relative to uninduced MT1110. Error bars represent standard error for biological replicates (*n* = 3 ** highly significant difference, one way ANOVA, *F* = 11.96, p = <0.01).

To confirm that the asRNA was reducing actinorhodin biosynthesis through a transcript silencing mechanism, RT-PCR and qRT-PCR were employed to monitor expression of *act*I-ORF1 mRNA (Fig. 5). RT-PCR analysis confirmed earlier experiments, with only induced MT1110/pAS01 showing a decrease in the abundance of *act*I-ORF1 mRNA. Primers targeting the 5′ end of the transcript yielded less amplification product than those hybridising towards the 3′ end (Fig. 5A). Assuming equal primer access and amplification kinetics, this is empirical evidence for the mRNA/asRNA hybrid being degraded in the 5′ –3′ direction triggered by hybridization of the asRNA. qRT-PCR analysis of replicate RNA extracts from 3-d-old cultures was carried out with SCO4742 as the internal reference and values were calculated relative to the expression levels of uninduced MT1110. Consistent with antibiotic assays and RT-PCR data, only induced MT1110/pAS01 showed a significant difference (≈ 5-fold decrease) in the level of *act*I-ORF1 expression (Fig. 5B).

### Conclusions/Concluding Remarks

In this study we have demonstrated that synthetic asRNA can be designed and used to regulate gene expression in *S. coelicolor*. Furthermore, RNA silencing was effective when expressed from either the chromosome or from a replicative plasmid. The application of antisense RNA silencing in the genus *Streptomyces* provides a complimentary tool to those of standard genetics and is applicable in instances where gene knockouts are not suitable (see above). This genus is responsible for the production of a large number of high value compounds and the development of techniques for improving titre through metabolic engineering are industrially significant [26]. Antisense RNA silencing is of particular value in the field of metabolic engineering since it can avoid problems associated with lethal mutations caused by gene knockouts; the level of silencing is variable and complete inhibition of protein production can be avoided [27], [28]. We applied RNA silencing to inhibit production of an antibiotic, however as earlier studies with natural asRNAs have shown [10], [11], it is possible that these methods could be used to induce expression of silent biosynthetic gene clusters by silencing repressors, or by interfering with mRNA secondary structures that sequester ribosome binding sites [1]. Comparison of the natural *mic*X asRNA with that of the synthetic asRNA designed for this study revealed possible areas for the future development of asRNAs for use in *Streptomyces*: The *mic*X asRNA has a predicted paired-end structure that contains regions of non-complementary nucleotides (Fig. 2); in *E. coli* these regions have been shown to protect antisense RNAs from RNase III cleavage [29]. Furthermore, as with other natural *trans*-encoded antisense RNAs, the mRNA binding region of *mic*X is ≈ 25 bp. The paired-termini approach used in this study has been applied successfully using mRNA binding regions <30 bp (Nobutaka Nakashima, AIST Sapporo, personal communication) and thus the design of synthetic asRNAs based on naturally occurring *trans*-encoded asRNAs may yield more effective gene silencers. It is also likely that a better understanding of the mechanism of antisense RNA gene silencing in *Streptomyces* will lead to improvements in asRNA design. For example, in many species, limited complementarity between natural *trans*-encoded asRNAs and the corresponding mRNA requires the RNA chaperone Hfq to facilitate RNA-RNA interactions [30]. Hfq is believed to protect asRNAs from degradation when they are not bound to their target mRNAs, and to recruit the RNA degradation machinery once the asRNA is paired to the target mRNA. [1] There are no obvious homologues of Hfq in the genome of *S. coelicolor* [9] and it remains to be determined whether an RNA chaperone is required for asRNA gene silencing in *Streptomyces*. Long stretches of base pairing, or high concentrations of the asRNA, as in the case of the synthetic asRNAs used in this study, may obviate a chaperone requirement [1]. However, scr5239, a *trans*-encoded sRNA repressor of extracellular agarase expression, has five stem-loop structures that presumably require facilitated opening in order for annealing to occur, leading the authors to speculate that *Streptomyces* species may require proteins to carry out the functions of Hfq [12]. Consequently, the identification of such an RNA chaperone in *Streptomyces* could aid in the development of synthetic asRNAs since synthetic RNAs could then be designed to mimic naturally occurring *trans*-acting sRNAs. The use of peptide-PNAs in *Streptomyces* is of particular interest as genetic transformation is not required for gene silencing, thus they can be used in species in which genetic tools have not been established or to simply accelerate the process of genetic analysis. The current synthesis costs and high concentrations required when using these molecules in agar-based assays makes optimization studies prohibitive. However, improvements in micro-culture of *Streptomyces* [31] and reduced synthesis costs will enable different peptide-PNA designs to be evaluated for this genus. In summary, recent reports reveal that *Streptomyces* use endogenous transcripts to regulate gene expression, and here we show for the first time that synthetic strategies using expressed RNA or a delivered DNA mimic can provide useful levels of RNA silencing. As a method, RNA silencing can be used to improve our understanding of *Streptomyces* biology and possibly alter metabolic flux in industrial applications.

## Materials and Methods

### Bacterial Strains, Plasmids and Culture Conditions

The strains and plasmids used in this study are listed in Table 1. Standard media and conditions for growth were as previously described [24], [32]. The media were supplemented with the following chemicals when required: 100 µg/ml ampicillin (Amp), 50 µg/ml kanamycin (Kan), 25 µg/ml chloramphenicol (Chl), 25 µg/ml nalidixic acid (Nal), 50 µg/ml apramycin (Apr), 25 (or 50) µg/ml thiostrepton (Tsr) and 0.1% (wt/vol) ε-caprolactam. Growth in liquid R5 was monitored by optical density (A_430_).

**Table 1:** Bacterial Strains and plasmids used in this study.

Strain or plasmid	Description	Source or reference
*Streptomyces coelicolor* MT1110	Wild-type prototrophic; SCP1^−^, SCP2^−^ derivative of *Streptomyces* *coelicolor* A3(2)	[24]
*Escherichia coli* DH5α	For cloning and propagation of host strain, *recA1* endA1 *gyrA96 thi-1* *hsdR17 supE44 relA1 lac* [F’ *proAB lacI* ^q^ *ZΔM15* Tn*10* (*Tet^r^*)]	Stratagene
*E. coli* ET12567	*dam dcm hsdS hsdR* Chl^r^ Kan^r^; carrying plasmid pUB307	[39]
*Bacillus mycoides* ATCC 6462	As an indicator organism for bioassay of calcium-dependent antibiotic	American Type Culture Collection
pGEMT-Easy	Amp^r^; *E. coli* vector for cloning PCR products	Promega
pUB307	RP1 derivative contains *ori*T from IncP-group plasmid RK2	[39]
pIJ8600	Apr^r^ Tsr *tip*Ap; thiostrepton inducible integrative expression vector	[24]
pSH19	Tsr^r^ *Pnit*A; replicative expression vector	[25]
pAS01	Apr^r^ Tsr^r^; 155 bp (-92 to +63) of *act*I-ORF1. Cloned in antisense orientationinto pIJ8600	This study
pAS02	Apr^r^ Tsr^r^; 155 bp (-92 to +63) of *act*I-ORF1. Cloned in antisense orientationinto pSH19	This study

### Antibiotic Assays

Spores (3×10^6^ colony forming units [CFU]) of the experimental and control strains were inoculated into 20 ml of R5 liquid medium containing the appropriate antibiotics to an OD_450_ of ≈ 0.05. Cultures were grown for 18 h before 1 ml was transferred to 50 ml of R5 liquid medium containing appropriate antibiotics and inducers (for induction samples). Actinorhodin and undecylprodigiosin samples from three biological replicates were quantified according to standard protocols [24], [33]. Production of the calcium-dependent antibiotic was assayed using *Bacillus mycoides* ATCC 6462 as an indicator strain [34].

### Peptide Nucleic Acid (PNA) RNA Silencing of actI-ORF1

Sc001 and Sc002, antisense PNAs specific for *act*I-ORF1, were designed using previously described parameters [16], [35] and differed in their degree of coverage of the start codon of *act*I-ORF1. A scrambled PNA that had no sites of perfect complementarity in the genome of *S. coelicolor* MT1110 was used as a negative control (see Table 2 for PNA sequences). The cell-wall permeating peptide (KFF)_3_K, was attached to the N-terminus of each PNA (which corresponds to a 5′ nucleic acid terminus) via a flexible ethylene glycol linker [16]. Peptide-PNAs were synthesized as previously described [14]. Fifty microlitres of a *S. coelicolor* MT1110 spore suspension (1.5×10^8^ CFU/mL) were used to prepare a confluent lawn of growth (24 h incubation at 28°C) on MPCA agar plates (yeast extract, 0.2%, meat extract 0.2%, Bacto-peptone 0.4%, NaCl 0.5%, MgSO_4_.7H_2_O 0.2%, glycine 0.5%, glucose 1.0%, sucrose 10%, casamino acid 0.01%, TES buffer 0.573% and agar 2%). Peptide-PNAs were applied directly to the lawn at concentrations of 1, 10 and 50 µM and incubation continued at 28°C for 72 hrs. To determine whether inhibition of actinorhodin production was due to RNA silencing of *act*I-ORF1 or to non-specific toxicity of the PNAs, subcultures from the zones of application were inoculated onto MPCA agar plates. ISP-4 agar (inorganic salts-starch; Difco) that supports both undecylprodigiosin and actinorhodin production in *S. coelicolor* MT1110 was used to evaluated the specificity of gene silencing: agar plates were overlaid with 0.5% ISP-4 agar containing 2.3×10^6^ CFU/mL *S. coelicolor* MT1110 spores, 10 µL of PNAs (50 µM) were applied to antibiotic assay discs (Whatman) and placed onto the soft agar. After 96 hours of incubation, the disks were removed and the agar plates were photographed.

**Table 2:** Primers and peptide-PNAs used in this study.

Oligomer^a^	Sequence 5′–3′	Description
actIORF1258f	gccctaccgttcacaggtc	RT-PCR of *act*I-ORF1
actIORF1497r	tccgacagcagcagatactc	RT-PCR of *act*I-ORF1
actIORf1860f	tcgtcctggaggactacgac	RT-PCR and qRT-PCR of *act*I-ORF1
actIORF11148r	atcgagctgaccggagtg	RT-PCR and qRT-PCR of *act*I-ORF1
SCO4742_338f	gttccgccgaggagttgat	RT-PCR and qRT-PCR of SC04742 (internal control)
SCO4742_730r	gccggtacttgtcgctctc	RT-PCR and qRT-PCR of SC04742 (internal control)
actIPT1HindSph-F	gcg**caagctt**CAGTGGTGGTGGTGGTGGTGC**gcatgc**aggctcgaaggccgatac	PCR of paired-termini antisense *act*I-ORF1 for cloning into pIJ8600
actIPT1EcoRPst-R	cgg**gaattc**CAGTGGTGGTGGTGGTGGTGC**ctgcag**ctgcgtggccatgtgttc	PCR of paired-termini antisense *act*I-ORF1 for cloning into pIJ8600
actIPT1NdeSph-F	gcgc**catatg**CAGTGGTGGTGGTGGTGGTGC**gcatgc**aggctcgaaggccgatac	PCR of paired-termini antisense *act*I-ORF1 for cloning into pSH19
actIPT1XbaPst-R	cgg**tctaga**CAGTGGTGGTGGTGGTGGTGC**ctgcag**ctgcgtggccatgtgttc	PCR of paired-termini antisense *act*I-ORF1 for cloning into pSH19
Peptide-PNA^b^		
Sc001	(KFF)_3_K-eg1-gcaccccat	Antisense RNA silencing of *act*I-ORF1– binds -5 to +4 relative to start codon
Sc002	(KFF)_3_K-eg1-ccccatctc	Antisense RNA silencing of *act*I-ORF1– binds −8 to +1 relative to start codon
Scramble	(KFF)_3_K-eg1-ccatttagtt	Control PNA

a Restriction sites are in bold, paired termini sequences are in capitals.

b eg1 = ethylene glycol linker.

### Construction of Antisense RNA Expressing Vectors

A 155 bp fragment (−92 to +63 bp of the *act*I-ORF1 gene) was amplified from *S. coelicolor* MT1110 gDNA using primers modified to include paired-termini sequences [20]. Primers actIPT1HindSph-F and actIPT1EcoRPst-R were used for cloning into pSH19. Primers actIPT1NdeSph-F and actIPT1XbaPst-R were used for cloning into pIJ8600 (see Table 2 and Figure S1). Primers contained *Sph*I and *Pst*I restriction sites for convenient excision and replacement of the antisense RNA expressing fragment of the vectors.

### DNA Cloning and Bacterial Transformation and Streptomyces Conjugation

DNA manipulations, *E. coli* transformations and *Streptomyces* conjugations were carried out according to standard protocols [24], [32].

### RNA Extraction from Streptomyces Strains

RNA was extracted from *Streptomyces* strains grown in liquid R5 medium (as above) using a modified Kirby mix, phenol/chloroform extraction and DNAse I treatment [24]. RNA samples were further purified using RNeasy columns according to the manufacturer’s instructions (Qiagen, UK).

### RT-PCR and qRT-PCR

RT-PCR was performed using the Promega Access RT-PCR system with 400 ng RNA per sample. The RT-PCR conditions were as follows: for cDNA synthesis, 45°C for 45 min, followed by 94°C for 2 min. For PCR amplification, 20 cycles at 94°C for 30 s, 55°C for 1 min, and 68°C for 2 min and 1 cycle at 68°C for 10 min. Two primer pairs (actIORF1258f/actIORF1497r and actIORf1860f/actIORF11148r) were used to amplify distinct regions of the *act*I-ORF1 mRNA. SCO4742 a hypothetical gene that shows little variation in expression [36], [37] was used as an internal reference (see Table 2 for primer sequences). Negative control experiments were run for each pair of primers in the absence of reverse transcriptase (but with DNA polymerase present). qRT-PCR was done using a Brilliant II SYBR® Green QRT-PCR Master Mix 1-step kit (Agilent). Amplification and analysis were performed on a Bio-Rad iCycler. Each 20-µl reaction contained 500 ng RNA template, 1 µM each of actIORf1860f and actIORF11148r, 10 µl 2x Master mix and 1 µl of reverse transcriptase enzyme mix (containing RNase inhibitor). The qRT-PCR conditions were as follows: 1 cycle of 50°C for 30 min (reverse transcription), followed by another 1 cycle of 95°C for 10 min (to activate the DNA polymerase), then 40 cycles of 95°C for 30 s, 50°C for 30 s, 72°C for 1 min. An additional 1 cycle at 72°C for 5 min was performed. SCO4742 was used as the internal standard. Estimation of the relative amounts of transcript in each sample and the statistical calculations were as described [34] with the following modifications: a standard curve was obtained with a set of sonicated serially diluted genomic DNA positive controls. The amplification efficiency was calculated by taking the gradient of the line of best fit through plots of log_10_ dilution factors against the C_t_ values. The efficiency was corrected based on multiple samples [38]. Negative controls were as above.

## Supporting Information

Figure S1 **Schematic representation of the cloning strategy used to construct antisense RNA expressing vector pAS02.**
(TIFF)

Figure S2 **Calcium-dependent antibiotic assay.** The assay was performed as previously reported [34] using *B. mycoides* as the indicator strain (A) wild-type *S. coelicolor* MT1110. (B) MT1110/pAS01 induced by adding thiostrepton to the agar. Plates were photographed and half images from each strain were aligned to compare the size of the zone of inhibition.
(PNG)

Table S1 **Off-target matches in** ***S. coelicolor*** **for PNAs used in this study.**
(DOCX)
