# Supplementary figures and images for: Synthetic RNA Silencing of Actinorhodin Biosynthesis in Streptomyces coelicolor A3(2)
Source: PLoS One. 2013 Jun 27;8(6):e67509. doi: 10.1371/journal.pone.0067509 (PMC3694883; doi:10.1371/journal.pone.0067509)

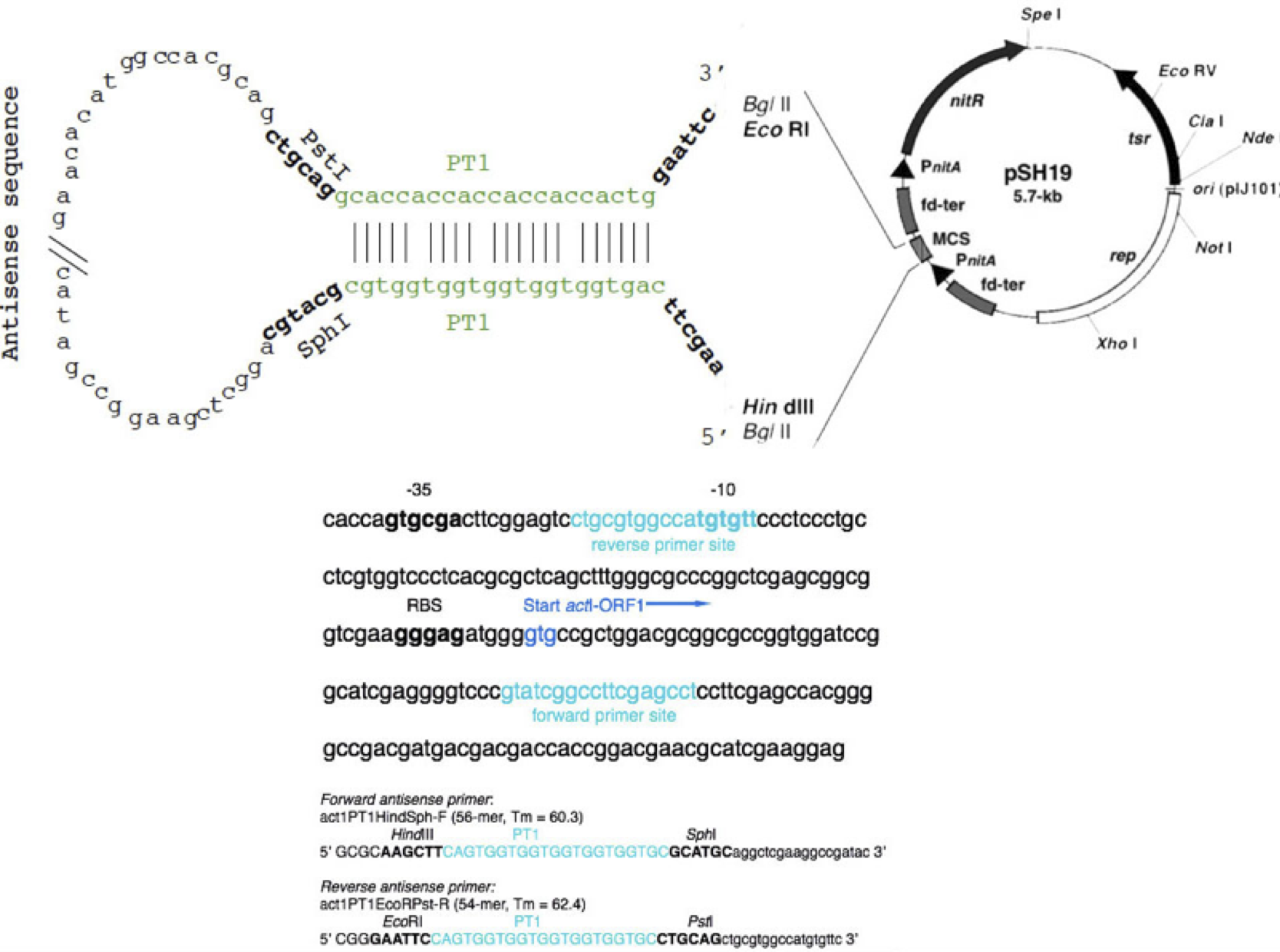

Supplement: Figure S1 — Schematic representation of the cloning strategy used to construct antisense RNA expressing vector pAS02. (TIFF) [file pone.0067509.s001.tiff]

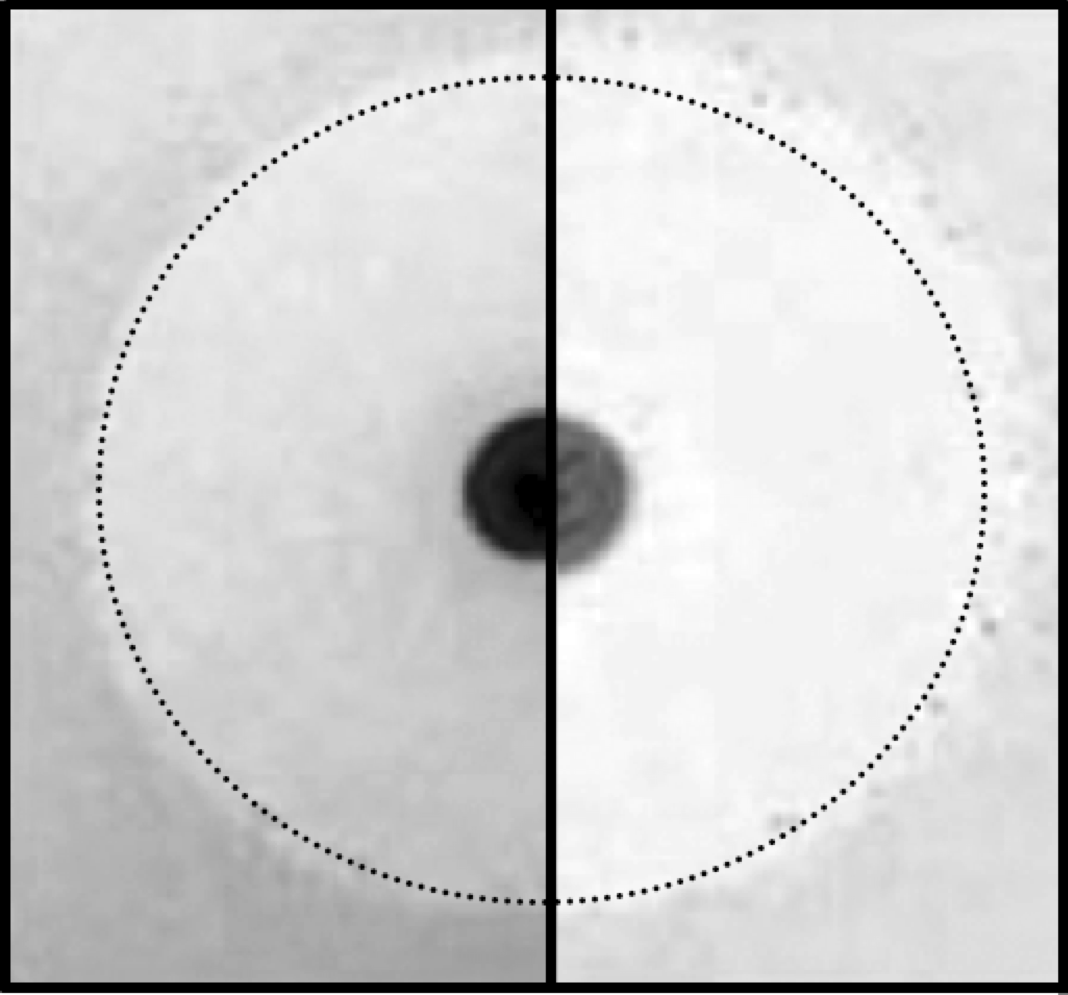

Supplement: Figure S2 — Calcium-dependent antibiotic assay. The assay was performed as previously reported [34] using B. mycoides as the indicator strain (A) wild-type S. coelicolor MT1110. (B) MT1110/pAS01 induced by adding thiostrepton to the agar. Plates were photographed and half images from each strain were aligned to compare the size of the zone of inhibition. (PNG) [file pone.0067509.s002.png]
