# Supplementary material for: Synthetic RNA Silencing of Actinorhodin Biosynthesis in Streptomyces coelicolor A3(2)
Source: PLoS One. 2013 Jun 27;8(6):e67509. doi: 10.1371/journal.pone.0067509 (PMC3694883; doi:10.1371/journal.pone.0067509)
Supplement: Table S1 — Off-target matches in S. coelicolor for PNAs used in this study. (DOCX) [file pone.0067509.s003.docx]

| **PNA** | **Off-target* matches** | | **No. of mismatches (bp)** |
| --- | --- | --- | --- |
|  | **Gene** | **Predicted function** |  |
| Sc001 | SCF62.15 | possible lipoprotein | 1 |
|  | SCM2.18c | possible integral-membrane protein | 1 |
|  | SCI7.13c | probable dihydropicolinate synthase | 1 |
|  | SCD25.24c | unknown | 1 |
|  | SCD63.10 | *pka*I, serine/threonine protein kinase | 1 |
|  | SC8A11.15c | possible lipoprotein | 1 |
|  | SC5H1.12 | *clp*P4, probable ATP-dependent Clp protease | 1 |
|  | SC8D11.15c | hypothetical protein | 1 |
|  | SCBAC1A6.16 | conserved hypothetical protein | 1 |
|  | SC4A2.12 | probable integral membrane protein | 1 |
|  | SCBAC1A6.16 | conserved hypothetical protein | 1 |
|  | SC8E7.01 | possible membrane protein | 1 |
|  | 2SCI34.06 | possible integral membrane protein | 1 |
|  | SCI7.02c | unknown | 1 |
|  | SCC61A.01 | hypothetical protein | 1 |
|  | SC1A6.10 | probable two-component system sensor kinase | 1 |
|  | 2SCG61.30c | unknown | 1 |
|  | SCD95A.01 | unknown | 1 |
|  | SCD40A.10c | possible membrane protein | 1 |
| Sc002 | SC4C6.13c | *pca*I, probable 3-oxoadipate CoA-transferase subunit A | 0 |
|  | SC4B10.21 | *qcr*B2, ubiquinol-cytochrome C reductase cytochrome B | 1 |
|  | SCF62.16 | possible membrane protein | 1 |
|  | SCF55.27 | probable amino transferase | 1 |
|  | SCD31.40 | *rps*N, 30S ribosomal protein S14 | 1 |
|  | SC1A8A.11 | phosphoenolpyruvate-protein phosphotransferase | 1 |
|  | SCI51.20c | possible DNA repair protein | 1 |
|  | SC4G10.14c | *pep*1B, possible alpha-amylase | 1 |
|  | SCJ33.05c | possible calcium-binding protein | 1 |
|  | SCI11.12c | possible RNA polymerase sigma factor | 1 |
|  | SC4A10.29 | possible membrane protein | 1 |
|  | SCC30.02c | probable *ara*C-family regulatory protein | 1 |
|  | SCC24.13c | probable *gnt*R-family transcriptional regulator | 1 |
|  | SCAH10.12 | possible allantoinase | 1 |
|  | SCG11A.25 | probable export protein | 1 |
|  | SC10A9.18 | probable *lac*I-family transcriptional regulatory protein | 1 |
|  | SC1A8A.22c | *cvn*A4, possible large secreted protein | 1 |
|  | SCI35.18c | Probable two-component sensor | 1 |
|  | SCE20.24c | possible 2-hydroxyhepta-2,4-diene-1,7-dioate isomerase | 1 |
|  | SCH24.16c | probable transmembrane protein | 1 |
|  | SCD65.07c | probable hydrolase | 1 |
|  | SC5F2A.13 | possible racemase | 1 |
|  | SC2H2.13 | possible DNA-binding protein | 1 |
|  | SCF51.14 | possible integral membrane protein | 1 |
|  | SCE15.10c | possible small integral membrane protein | 1 |
|  | SCH63.17 | probable cytochrome P450 oxidoreductase | 1 |
|  | SC5H1.12 | *clp*P4, probable ATP-dependent Clp protease 2 | 1 |
|  | SCF51.13c | possible RNA polymerase sigma factor | 1 |
|  | 2SCG2.13c | integrase | 1 |
|  | SCL2.19 | possible membrane protein | 1 |
|  | SCL24.11 | possible thiamine biosynthesis lipoprotein precursor | 1 |
|  | SCAH10.02 | possible regulatory protein | 1 |
|  | SCC121.22c | probable membrane protein | 1 |

* Genes with a transcriptional initiation region with ≤ 1 bp mismatch with the intended PNA target sequence
